# Detailed Radiomorphometric Analysis of the Surgical Corridor for the Suprageniculate Approach

**DOI:** 10.3390/jpm14050516

**Published:** 2024-05-12

**Authors:** Tomasz Wojciechowski, Nicola Bisi, Kazimierz Szopiński, Daniele Marchioni

**Affiliations:** 1 Department of Descriptive and Clinical Anatomy, The Medical University of Warsaw, 5 Chalubinskiego St., 02004 Warsaw, Poland; tomasz.wojciechowski@wum.edu.pl; 2 Department of Otorhinolaryngology, Head and Neck Surgery, The Medical University of Warsaw, 1a Banacha St., 02097 Warsaw, Poland; 3 Department of Otorhinolaryngology—Head and Neck Surgery, Azienda Ospedaliero-Universitaria di Modena, Via del Pozzo 71, 41125 Modena, Italy; daniele.marchioni@unimore.it; 4 Department of Dental and Maxillofacial Radiology, The Medical University of Warsaw, 6 Bienieckiego St., 02097 Warsaw, Poland; kazimierz.szopinski@wum.edu.pl

**Keywords:** suprageniculate approach, suprageniculate fossa, geniculate ganglion, endoscopic ear surgery

## Abstract

Background: The suprageniculate fossa (SGF) is located between the geniculate ganglion, the middle cranial fossa (MCF) and the anterior semicircular canal (ASCC). An endoscopic transcanal approach has been recently proposed to treat the different lesions in this area. The aim of the study is to describe the anatomical pathway of this approach by measuring the dimensions of its boundaries while checking their correlation with the pneumatization of the SGF area. Methods: This is a retrospective anatomical analysis of Cone Beam CT scans of 80 patients, for a total of 160 temporal bones analyzed. Two checkpoints were measured for the SGF route, as an internal and an external window. These are triangles between the MCF dura, the geniculate ganglion and the ASCC on parasagittal and axial planes. The pneumatization of the SGF was also assessed, classified and correlated with the measured dimensions. Results: The depth of the SGF was 7.5 ± 1.8 mm. The width of the external window was 7.5 ± 1.9, 5.6 ± 2.4 and 1.6 ± 1.6 mm for the posterior, middle and anterior points of measurement, respectively. The height of the internal window was 7.6 ± 1.2, 4.5 ± 1.5 and 1.7 ± 1.7 mm for the posterior, middle and anterior points of measurement, respectively. Type A pneumatization was found in 87 cases, type B in 34 and type C in 39. The degree of pneumatization directly correlated to the depth and height of the fossa. Conclusions: The suprageniculate approach route is defined by the internal and external windows which should be evaluated during a pre-surgery imaging assessment. The detailed anatomy of the approach and the novel classification of the pneumatization of the SGF are here described which may be useful to plan a safer procedure with minimal complications.

## 1. Introduction

The suprageniculate fossa (SGF) is an irregular pyramidal-shaped space located at the boundaries between the middle ear, the lateral skull base and the middle cranial fossa. Pathological processes involving this particular area are diverse but quite rare [1]. They include both inflammatory diseases and tumors, i.e., chronic otitis media with cholesteatoma, meningiomas, facial nerve ossifying hemangiomas, schwannomas, neurofibromas, epidermoid cysts, choristomas, and rarely metastases [2,3].

The main symptoms of SGF lesions are progressive facial weakness, recurrent paresis or even complete paralysis. Depending on the lesion’s extension and its involvement in the ossicles or otic capsule, conductive, sensorineural or mixed hearing loss may also occur. One must bear in mind that often, SGF diseases appear with normal hearing or facial function and symptoms might initiate in an advanced stage of the disease.

Surgery is the treatment of choice for most of the pathological processes close to the SGF with the exception of facial nerve neoplasms. While radiosurgery remains a possible treatment option [4,5], facial nerve tumors are usually managed with a “wait and scan” approach alone, at least when the facial nerve function is preserved at the time. In fact, surgery with the ultimate objective of complete tumor excision is reserved for patients with a grade III HB or worse. The reason is that while an early intervention could grant nerve preservation and the possibility to avoid progressive facial weakness, the tumor cannot always be separated from the nerve, resulting in nerve section and the need for graft reconstruction [6,7,8]. The hearing function is also an important aspect to consider in patients suffering from SGF lesions. Combining the information about these two groups of symptoms with the patient’s expectations may guide the decision-making process on the proper surgical approach when a surgical treatment is indeed needed.

In order to remove SGF lesions, extensive surgical procedures are traditionally performed. When there is a pre-operative serviceable hearing, the technique of choice is often the middle cranial fossa (MCF) approach. It may also be combined with a transmastoid procedure for lesions involving the middle ear and the mastoid, for example in the case of cholesteatoma with SGF extension. However, one has to take into account the possible complications of an MCF approach due to the extradural elevation and retraction of the temporal lobe in order to gain adequate exposure, i.e., temporal lobe injury, intracranial bleeding, cerebrospinal fluid leakage and postoperative seizures.

**Figure 1:**
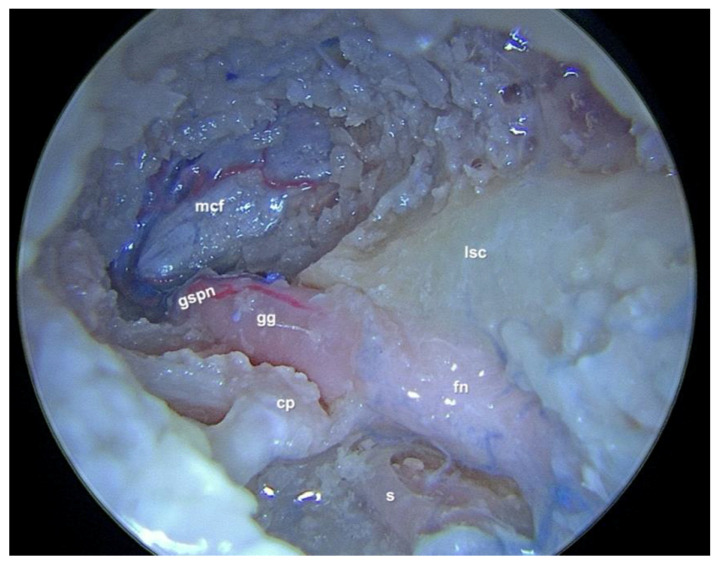
Cadaveric dissection of the left middle ear; the exposure of the suprageniculate fossa can be seen after the head of the malleus and whole incus removal, middle fossa dura exposure and tympanic facial nerve and geniculate ganglion decompression. cp: cochleariform process; s: stapes; fn: tympanic part of the facial nerve; lsc: lateral semicircular canal; gg: geniculate ganglion; gspn: greater petrosal nerve; mcf: middle cranial fossa dura.

The SGF as described by Marchioni et al. [9] is defined superiorly by the MCF dura, inferiorly by the geniculate ganglion and the beginning of the tympanic facial nerve, and posteriorly by the ampullated ends the lateral and anterior semicircular canals (Figure 1). The epitympanum serves as the lateral border of the pyramidal space, and the portion of the petrous apex (PA), located superiorly and anteriorly to the internal auditory canal (IAC), represents the medial limit.

The advancements in transcanal endoscopic ear surgery (TEES) and its growing application in lateral skull base [10] have enabled the surgeon to gain access to the SGF and to remove lesions from this location using the external auditory canal (EAC) as a minimally invasive pathway, without the need for a craniotomy or a temporal lobe retraction [9]. An exclusive transcanal endoscopic approach to lesions of the SGF is feasible when there is no mastoid involvement, nor any cochlear or intradural extension. The main disadvantage of the technique is the need for the incus and malleus head removal to expose the targeted area, often requiring a subsequent ossiculoplasty. Thus, the perfect situation for the approach is when the patient presents with pre-operative conductive hearing loss, ossicular chain erosion or profound sensorineural hearing loss, presenting an SGF pathological process [9].

It is common to describe surgical approaches by establishing straightforward checkpoints and to propose step-by-step dissection to easily reproduce and teach them. Drawing inspiration from the work of Cömert et al. on the cadaveric surgical anatomy of the infralabyrinthine approach and their definition of an access window and an internal window to the petrous apex [11], we set out to analyze the data from radiologic studies, define the possible problems and determine whether it is possible to perform an approach according to different levels of pneumatization. The radiomorphometric studies also serve as a link between theory and clinical practice as they provide information not only for surgeon’s visualization but also for the development of new tools that may be used.

The aim of the study was to define a surgical corridor for the transcanal endoscopic approach to the SGF (1), to calculate the dimensions of the corridor checkpoints on CBCT scans of adult patients (2), to correlate the pneumatization of the SGF and its measurements in the analyzed scans for a better understanding of the preoperative situation and planning (3), and to propose a new classification of the pneumatization around the geniculate ganglion (4).

## 2. Materials and Methods

This retrospective study was performed on anonymized sets of Cone-Beam Computed Tomography (CBCT) images of the cranium gathered from the Department of Dental and Maxillofacial Radiology, Medical University of Warsaw between February 2016 and June 2018. All the scans belong to adult patients and were obtained due to dental or maxillofacial indications with a Planmeca Promax 3D Mid CBCT scanner (Planmeca USA, INC, Roselle, IL, USA), with a source voltage of 90 kV, a current of 12 mA and voxel dimensions of 400 × 400 × 400 µm.

In total, 80 sets of scans (40 males and 40 females) were enrolled in the study and thus 160 temporal bones were analyzed. In total, 80 CBCT scans were included in the study of 40 males and 40 females with an age range spanning from 17 to 84 years (mean value 36.44 and SD 13.14). Every considered temporal bone had a well-pneumatized mastoid (groups 3 and 4), according to Tan et al.’s classification [12]. The scans that showed temporal bone pathologies (developmental alterations of the bony labyrinth, fractures or effusion in the middle ear spaces) were excluded from the study. All scans were analyzed using the RadiAnt DICOM Viewer 2022.1 (64-bit; Medixant, Poznan, Poland) software. The window level was set to 500 Hounsfield Units (HU) and the window width to 3500 HU.

**Figure 2:**
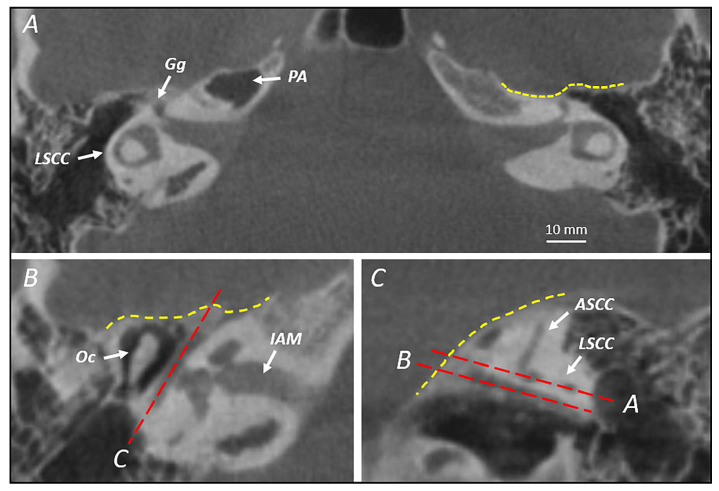
(**A**) Symmetrized horizontal plane of bilateral “signet ring” appearance of the lateral semicircular canal; (**B**) symmetrized horizontal plane at the level of the tympanic segment of the facial nerve, through which the tilted parasagittal plane is established (dashed red line); (**C**) tilted parasagittal plane where the two previously described tilted horizontal planes passing through the lateral semicircular canal and the tympanic facial nerve are shown (dashed red line). Yellow dashed line: irregular plane of middle cranial fossa dura; LSCC: lateral semicircular canal; Gg: geniculate ganglion; PA: petrous apex; Oc: ossicular chain; ASCC: anterior semicircular canal; IAM: internal acoustic meatus.

The first step of the images’ assessment was establishing a proper, reliable and repeatable protocol for the measurements. Each set of images was symmetrized on the tilted horizontal plane in order to see the classic “signet ring” appearance of the lateral semicircular canal (LSCC) on both sides. This represented the first measurement plane. Then, a variation in the parasagittal plane passing through the tympanic portion of the facial nerve was conceived as the second reference plane (Figure 2).

**Figure 3:**
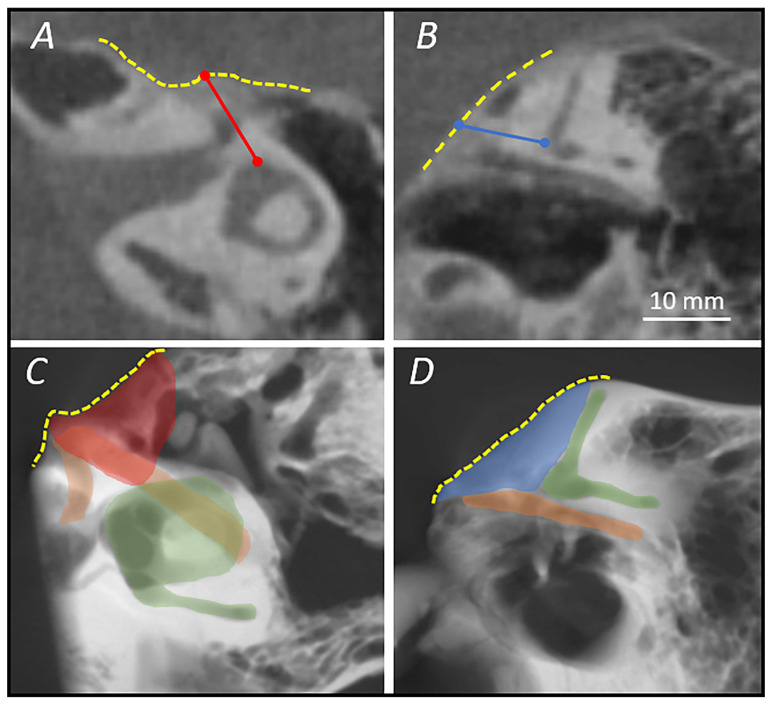
(**A**) Symmetrized horizontal plane where the depth of the fossa (D1) is shown as the distance between the anterior semicircular canal ampulla and the dural line; (**B**) tilted parasagittal plane where the depth of the fossa is shown (D2), only if D1 and D2 corresponded the following width and height calculations were performed; (**C**,**D**) micro-CT with average intensity projection of both panels (**A**,**B**), with the representation of the “external window” (red) and the “internal window” (blue) of the surgical corridor. Yellow dashed line: plane of MCF dura; orange area: facial nerve; green area: semicircular canals and vestibule.

The first measurement that was carried out was the depth of the suprageniculate fossa. On the symmetrized axial plane, the depth was defined as the distance between the ampulla of the ASCC and the MCF dura in the direction of the tilted parasagittal plane (D1). Conversely, on the tilted parasagittal plane, the depth of the fossa was measured as the distance between the ampulla of the ASCC and the MCF dura in the direction of the symmetrized axial plane (D2). This double check of the same fossa depth allowed us to verify the correct planes’ orientation, and only when D1 and D2 were equal did we set out to record the width measurements on the symmetrized axial plane and the height measurements on the tilted parasagittal plane, being confident that the starting point of both would be the same (Figure 3).

**Figure 4:**
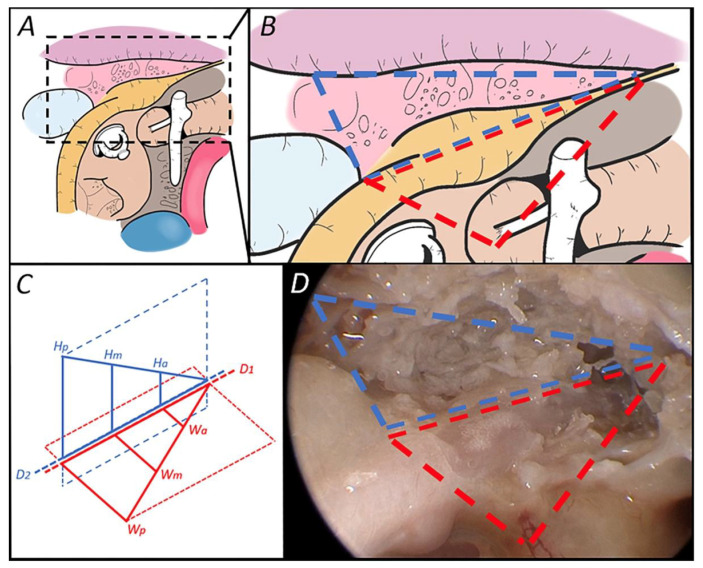
(**A**,**B**) Drawings of the suprageniculate fossa transcanal approach and its magnification with the representation of both the “external window” (red) and of the “internal window” (blue) of the approach. (**C**) Drawing of the access windows and their relative measurements of height (Hp, Hm and Ha), width (Wp, Wm and Wa) and depth (D2 and D1) on the tilted parasagittal and symmetrized horizontal planes. (**D**) Right ear of a cadaveric dissection where the access windows lines can be seen.

The width of the surgical access we defined as the distance between the facial nerve bony canal and the middle cranial fossa on the symmetrized axial plane, while the height of the approach was considered to be the distance between the tympanic facial nerve or geniculate ganglion and the middle fossa on the tilted parasagittal plane (Figure 4).

**Figure 5:**
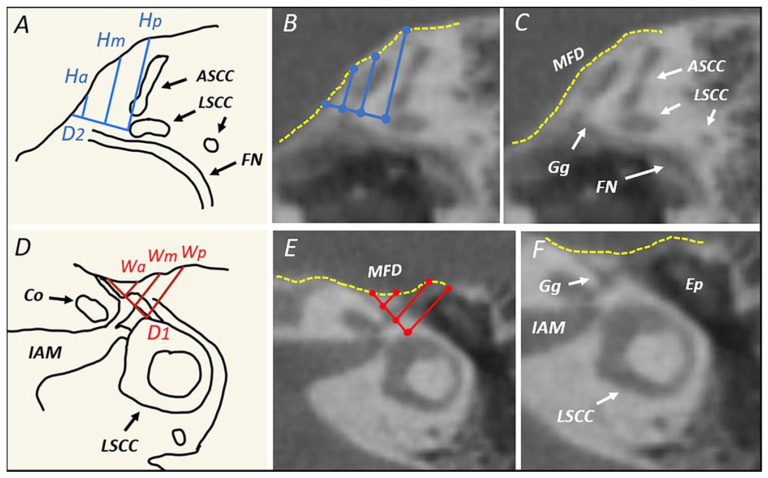
(**A**) Drawing of the tilted parasagittal plane with its anatomical landmarks and the height (Hp, Hm and Ha) and depth (D2) measurements; (**B**,**C**) CBCT of the same plane with measurements and anatomical landmarks, respectively; (**D**) drawing of the symmetrized horizontal plane with its anatomical landmarks and the width (Wp, Wm and Wa) and depth (D1) measurements; (**E**,**F**) CBCT of the same plane with measurements and anatomical landmarks, respectively. Yellow dashed line: plane of MCF dura; ASCC: anterior semicircular canal; LSCC: lateral semicircular canal; FN: facial nerve; IAM: internal acoustic meatus; Gg: geniculate ganglion; MFD: middle fossa dura; Ep: epitympanum.

For both width (W) and height (H), 3 series of measurements were performed (Figure 5):

- A posterior one (Wp and Hp) starting from the anterior end of the ampulla of the ASSC;
- A middle one (Wm and Hm) starting from the most posterior portion of the geniculate ganglion or from the angle between the labyrinthine and tympanic segments of the facial nerve;
- An anterior one (Wa and Ha) starting from the anteriormost point of the geniculate ganglion.

**Figure 6:**
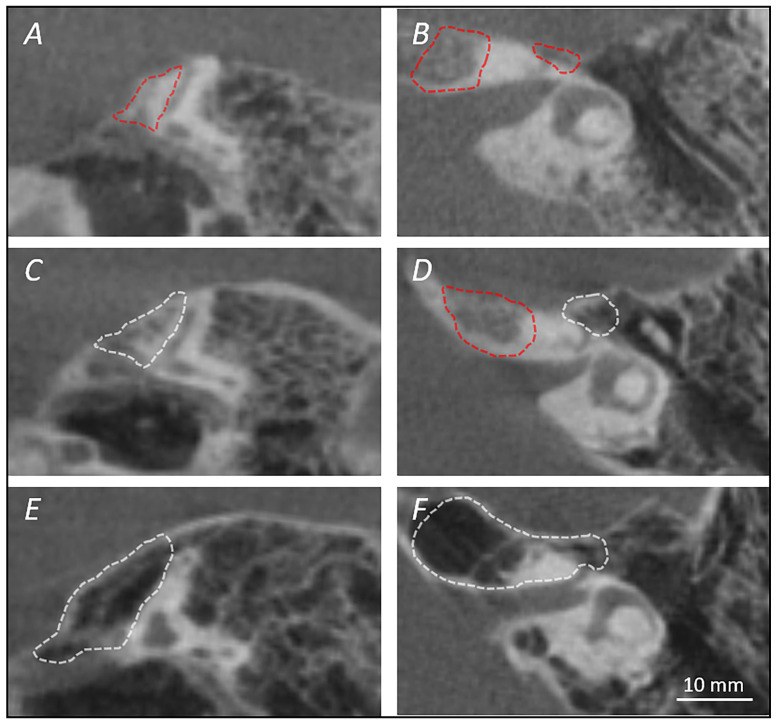
The different degrees of pneumatization on the tilted parasagittal plane on the left and on the symmetrized horizontal plane on the right. (**A**,**B**) Grade 0 (type A): no evidence of pneumatization over the geniculate ganglion and in the petrous apex; (**C**,**D**) grade 1 (type B): pneumatization of the air cells above the geniculate ganglion but no evidence of petrous apex pneumatization; (**E**,**F**) grade 2 (type C): evidence of pneumatization above the geniculate ganglion and at the level of the petrous apex. Red dashed line: no pneumatization present; white dashed line: evidence of pneumatization.

Finally, the pneumatization of the SGF was analyzed on both the symmetrized axial and the tilted parasagittal planes. The pneumatization was recorded for the bone area that lies anterior to the ASSC and superior to the geniculate ganglion, and thus three groups were established (Figure 6):

- Group 0 (type A): no evidence of pneumatization;
- Group 1 (type B): pneumatization of the air cells above the geniculate ganglion;
- Group 2 (type C): evidence of pneumatization above the geniculate ganglion and at the level of the petrous apex.

The obtained data were statistically analyzed using the IBM SPSS Statistics 25 software. For each of the measured parameters, the Shapiro–Wilk test was used to verify the normality of distribution, and then the following descriptive statistics were calculated: the mean value ± standard deviation (SD) and the min–max range. All parameters were compared according to the side, patient gender and pneumatization group (as previously explained). The observed differences were tested with Student’s *t*-test and a Chi-squared test. Pearson’s r correlation coefficient or Spearman’s rank correlation coefficient were additionally used. The *p*-value < 0.05 was considered significant for all comparisons.

The use of the CBCT scans for these temporal bone measurements was approved by the Ethics Committee of the Medical University of Warsaw (decision number AKBE 328/2023) and abides by the 1964 Helsinki Declaration and its later amendments or comparable ethical standards.

## 3. Results

### 3.1. Basic Descriptive Statistical Analysis of the Quantitative Variables

The overall depth of the SGF was 7.3 ± 1.8 mm (min–max: 3.3–12.7, measured on the symmetrized axial plane) and 7.5 ± 1.8 mm (3.5–12.8, measured on the tilted parasagittal plane). The width of the external window was 7.5 ± 1.9 mm (3.1–14.0) in the posterior, 5.6 ± 2.4 mm (0–11.4) in the middle and 1.6 ± 1.6 mm (0–8.5) in the anterior point of measurement. The height of the internal window was 7.6 ± 1.2 mm (4.3–11.2) in the posterior, 4.5 ± 1.5 mm (0–9.7) in the middle and 1.7 ± 1.7 mm (0–9.6) in the anterior point of measurement. The basic descriptive statistics of the studied variables were calculated according to the side and it is reported in Table 1. Shapiro–Wilk tests were calculated in order to verify the normality of the distributions of the considered quantitative variables. A close-to-normal distribution was observed for most of the variables. Only Wa (anterior width on the symmetrized axial plane) and Ha (anterior height on the tilted parasagittal plane) showed a different distribution from the Gaussian one, for both the right and the left side. The skewness of these last distributions was subsequently checked and, since the skewness values ranged between +/− 2, we assumed that the distributions of the Wa and Ha values were not significantly asymmetric if compared to the mean value. It was thus decided to use parametric tests for the subsequent statistical analysis.

### 3.2. Height and Width of the Corridor and Patients’ Gender

Then, the access height and width were compared, still sub-divided by side, between the male subgroup and the female subgroups. To do so, the Student’s *t*-test for independent samples was used. Table 2 presents statistically significant differences that were found for depth measurements, both on the symmetrized horizontal plane (D1) and on the tilted parasagittal one (D2), for which the value was higher on the right side of the males. The dimension of the observed effect, measured with Cohen’s d, was moderately large. No further statistically significant differences were found.

### 3.3. Corridor’s Height and Width and Measurement Side

A further step implied the comparison between the access height and width values between the right side and left side subgroups. To do so, the Student’s *t*-test for dependent samples was used. As Table 3 shows in detail, no statistically significant differences were found in the measurements between the right and left sides.

### 3.4. Correlation between the Different Height and Width Measurements of the Corridor

The relationships between the different dimensions of the access height and width were then measured, through a series of correlation studies with Pearson correlation coefficient (r), separately for the right and left sides. Table 4 shows how the correlations were mostly statistically significant (bold in the table). Positive correlation indexes indicate a directly proportional increase in the associated dimensions. The intensity of the correlation turned out to be variable and was graphically represented in Table 4 with different color shades.

### 3.5. Descriptive Statistics of the Degree of Pneumatization and Correlation between the Different Access Height and Width Measurements and the Degree of Pneumatization

The subdivision of the temporal bones according to the different degrees of described pneumatization was 87 for grade 0 (type A), 34 for grade 1 (type B) and 39 for grade 2 (type C). No statistically significant difference was identified as far as the pneumatization was concerned in relation to the analyzed side (Chi-squared test 2.839 and *p* = 0.242) or in relation to the gender of the subject (Chi-squared test 0.7836 and *p* = 0.676). The relationship between the different dimensions of the height and width of the corridor and the degree of pneumatization found in the suprageniculate fossa region was also under investigation. A number of analyses were carried out using Spearman’s rank correlation coefficient (R), separately for the left and the right side. Statistically significant relationships were detected, as shown in Table 5. Positive correlation indexes indicate a directly proportional increase in the associated dimensions. The correlation intensity was variable and it was graphically represented in Table 5.

## 4. Discussion

The suprageniculate fossa is an irregular pyramidal space at the crossroads between the middle ear and the lateral skull base. Lesions may occur in this area but these are rare and difficult to manage. The most common pathologies are tumors like hemangiomas or schwannomas of the facial nerve and inflammatory diseases such as localized or extensive cholesteatomas. The transcanal endoscopic approach to the SGF area has been recently proposed by Marchioni et al. [9]. It differs from the traditional approaches like the middle cranial fossa or subtemporal approach due to its reduced invasiveness but it also has some drawbacks. The approach requires the removal of the whole incus and the head of the malleus. As a consequence of such an approach, an ossiculoplasty should be performed afterward, at least in patients with conductive hearing loss and serviceable hearing function before surgery [9].

Given that the SGF has recently been introduced as an anatomical concept and that it is located at the convergence of important anatomical structures, in this paper we set out to obtain a reproducible dimensional analysis of the surgical transcanal corridor leading to the SGF in the total absence of data on the subject in the literature. We have also proposed to divide the corridor into subspaces between so-called checkpoints or windows (external and internal ones).

The main result of our study is the demonstration of a dimensional decrease in width (external window) and height (internal window) measurements of the analyzed surgical corridor in a postero-anterior direction. These measurements, in both height and width, in relation to the dura of the MCF, are the very limits of the approach and they give a perspective to the surgeon regarding the tools needed to obtain a good working space.

Our findings support the evidence from the publications that have already analyzed the different conformations of the posterior to anterior tegmen slope. These have underlined variability and the importance of an exact conformation of the patient’s middle fossa floor during the surgical procedure [13].

Of equal importance was the creation of a simple and reproducible protocol for the dimensional analysis of the surgical corridor to the suprageniculate fossa. This involves the proposition of two inclined planes and of the measurement of two access windows. Performing three measurements for each dimension (width and height) granted us the possibility to understand the shape of each window and to make sure the depth measurement (D) was the same on both obtained planes (also statistically confirmed by the perfect linear correlation between D1 and D2 values). This enabled us to carry out the three described measurements for each plane from the same starting point. The “external window” allowed us to evaluate the space into which the endoscopic tools must be inserted to perform the dissection of the fossa through a transcanal approach. The “internal window” corresponds to the lateral limit of the SGF and it allowed us to analyze its dimensions in relation to the middle fossa floor and the extension of the pneumatization. Our proposition of triangular-shaped spaces is a well-established anatomical concept.

The data analysis did not yield statistically significant differences between the height and width measurements, comparing them between sides. No significant differences were found either in the dimensional comparison between males and females, except for the depth measurement on the right side in males. Relying on this, we think the final shape of the SGF is more related to the development of the pneumatization than to the gender or side.

Among the different measurements in almost all the possible combinations, correlations were found to be statistically significant showing a directly proportional increase in pairs with varying degrees of intensity. This may help conceive the SGF as a cone-shaped area showing a sufficient working space above the ossicular chain, above the geniculate ganglion and medially to the epi- and protympanum. A significant element was the strong correlation between the posterior, middle and anterior height measurements (Hp, Hm and Ha) for the “internal window” and between those of the posterior, middle and anterior width (Wp, Wm and Ha) for the “external window”. It shows that the analyzed access windows both have a triangular morphology that remains consistent regardless of the absolute dimensional values. When the window is wide at the level of the ampullary end of the anterior semicircular canal, it is also wide at the level of the geniculate ganglion. The right way to interpret these dimensions is that, nearly always, we expect the posterior-most point of the height measurement to be very similar to the height of the ASSC. The middle and anterior-most points of measurement mirror the degree of inclination of the middle fossa on the sagittal plane (the so-called posterior-to-anterior slope). There may be situations in which the SGF is well pneumatized, the slope is mild and the geniculate ganglion is covered by bone, which is a safer anatomical configuration for the procedure to be performed.

It is also interesting to compare the width and the height measurements, where there is no significant correlation or there is a weak one, between Hp and Hm and Wp and Wm in all combinations. On the contrary, there are stronger correlations between Ha and all the width measurements and between Wa and all the height measurements. The maximum correlation intensity, however, is obtained by comparing Wa (mean 1.77 +/− 1.64 mm) and Ha (mean 1.7 +/− 1.56 mm). These two measurements are obtained starting from the anterior side of the geniculate ganglion, i.e., from the origin of the greater petrosal nerve. The dura of the middle cranial fossa runs in close proximity to the nerve at this level and the nerve or geniculate ganglion may be covered with bone of different thicknesses [14]. It is therefore possible to understand why variations in the level of the dura, in an area that is so close to the measurement point, may cause variations in the internal window anterior height and in the external window anterior width with an almost linear positive correlation between them.

A final correlation between the measurements to be considered is the ratio between the depth (D1 and D2), height and width measurements of the access windows. The depth was defined as the distance between the ampulla of the ASCC and the MCF, calculated in a direction parallel to the inclined parasagittal plane in the case of D1 and parallel to the symmetrized horizontal plane in the case of D2. This implies that D is dependent on how quickly the dural line lowers in an anterior direction (i.e., how far the posterior-to-anterior slope goes); thus, it is easy to understand how a deep suprageniculate fossa has a later anteriorly descending dura and therefore larger measures in the windows that have the same dural line as their limit.

Of extreme importance in our work was the evaluation of the degree of SGF pneumatization. We proposed a three-degree classification, depending on whether the air cells were not present, if they were present only at the level of the suprageniculate fossa or if they also extended to the level of the petrous bone apex. In about half of the analyzed temporal bones, no pneumatization was found (group 0 or type A), while the remaining ones were equally divided into the two remaining categories (group 1/2 or type B/C). No statistically significant difference was noticed regarding the pneumatization in relation to the side in which it was measured. This is in conformity with the findings of Tan et al., who verified a direct correlation between the pneumatization of the temporal bone on one side and the one on the other, even considering the single subgroup of analyzed air cells (mastoid, infralabyrinthine and of the apex) [12]. Furthermore, no difference was detected between the degree of pneumatization and the gender of the patients included in our study. On the other hand, Tan et al. found gender differences regarding the apex and infralabyrinthine pneumatization. Nonetheless, they concluded gender did not play a relevant role as far as mastoid pneumatization was concerned [12].

Where pneumatization was present above the geniculate ganglion, this positively correlated with most of the dimensional measurements. We saw that pneumatization was also correlated with the depth of the fossa and the value of the height measured at the posterior end of the geniculate ganglion (Hm). That suggests a deeper fossa and a greater height in its middle portion tend to be more pneumatized. Knowing the degree of pneumatization of the suprageniculate fossa has important surgical implications regarding the safety of the procedure, particularly during epitympanic cholesteatoma surgery. In fact, in the case of pneumatization, the epidermization can potentially extend to this region and in some cases even reach the apex, as it can happen in the case of a pneumatized subcochlear canaliculus [15]. We thus propose to use our new classification (types A, B and C) to assess the degree of pneumatization of the SGF and to check for the inflammatory process extension possibility.

It is worthy of note that a small group of patients had a pneumatization of the superior portion of the petrous apex, in the absence of suprageniculate fossa pneumatization. This could be an interesting starting point for future investigation; in fact, it would be important to precisely make out the origin of the pneumatization of the upper portion of the apex and whether or not this can be related to the pneumatization of the suprageniculate fossa.

Although the ultimate goal of this paper is a radiomorphometric investigation, there are some potential surgical implications for the data we obtained. Knowing the dimensions of the suprageniculate fossa and its degree of pneumatization before surgery enables the surgeon to adequately prepare to handle the pathology at hand. The measurement of what was described as the “external window” of the surgical route is of primary importance, as it determines what the operating space for the endoscopic instruments will be to then approach the suprageniculate fossa itself, whose lateral limit is represented by the “internal window”. We have demonstrated a correlation between the sizes of the two windows and this is true especially anteriorly. One may imagine a situation in which the SGF is high and pneumatized, but the access “external window” is narrow—this would make it very difficult to manage the lesion with an endoscopic transcanal approach.

Another important factor for surgical planning is knowing whether the lesion has extended to the petrous apex, following the pneumatization that connects the SGF to the apex itself. In this case, it is essential to drill the bone between the lateral semicircular canal, the dura of the middle fossa and the geniculate ganglion. Consequently, it becomes important to know the precise distance between these three structures before surgery, to be able to choose the suitable size of the drill to reach the apex region, keeping a safe distance from the anatomical landmarks. Some of the possible consequences of inadequate planning when working in this area might be the creation of an iatrogenic labyrinthine fistula, dural damage with a CSF fistula and facial paralysis [16]. From the data in our possession, we can deem the safest point to start the dissection to be at the level of the suprageniculate fossa just anterior to the ASCC, a level in which the distance among the structures is usually greater and so is the freedom of movement. In fact, moving anteriorly the dural line might descend very steeply, with the consequent risk of damaging it during the dissection.

The recent study by Molinari et al. [17] aimed to evaluate the clinical usefulness of the suprageniculate approach. The authors state that the exclusively endoscopic approach to the geniculate ganglion lesions may be an alternative to the traditional microscopic approaches in certain clinical situations and it may be expanded to reach the fundus of the internal acoustic meatus. They report 11 cases treated with this approach with no major complications and good facial nerve outcomes. In spite of the lack of other data in the literature, endoscopic ear surgeons may have to wait and see what place the suprageniculate approach will have in their armamentarium.

In the future, it would be interesting to evaluate on the basis of the data collected in this paper and with additional measurements carried out on subjects suffering from middle ear pathologies or with non-pneumatized mastoids, whether it is possible to define a few criteria to identify suitable patients for this transcanal access.

The main limitation of this retrospective study was the lack of anamnestic data of the patients whose Cone Beam CT scans were analyzed, in particular regarding any middle ear diseases. Furthermore, the analyzed group was limited to adult patients with well-pneumatized mastoids according to the classification proposed by Tan et al. [12]. This study therefore only offers a perspective on normal anatomy while this can be altered in the case of patients suffering from ear inflammatory diseases. Moreover, a measurement of the pneumatization of the suprageniculate fossa also in patients with diploic or sclerotic mastoids would be desirable to evaluate whether it is linked to the pneumatization of the entire temporal bone or not. As a final drawback, the comparison with the literature was limited; in fact, there is currently a lack of studies on the topic.

This anatomical study, based on CBCT scan analysis of a fair sample of adult subjects with well-pneumatized mastoids, enabled us to investigate in depth all the dimensional features of the transcanal surgical access corridor to the suprageniculate fossa through the creation of a standardized and reproducible protocol for the measurement of both an “external” and an “internal” window the surgeon must pass through while performing such an approach.

Although no differences were found in the measurements related to the analyzed side or to the gender of the patient under observation, statistically significant dimensional correlations were found with a high degree of intensity within the measurements of both windows, between their dimensions and the depth of the suprageniculate fossa, and between the anterior height and width of the approach.

A proposal for a novel radiological classification of the pneumatization of the SGF was also developed whose degree significantly correlates and is directly proportional in particular to the depth and height of the central portion of the SGF.

All these considerations can be used not only for descriptive purposes in order to better define the variability of the SGF area lying on the border between the middle ear and the lateral skull base. They can also provide clues to use the obtained results for the preoperative planning of endoscopic transcanal surgical approach or developing new tools as these minimally invasive procedures are the future of otology [18].

Further studies would be desirable to verify the results obtained in this paper, also in patients with chronic middle ear diseases and with poorly pneumatized mastoids. Also, correlating the pre-surgery measurements using our protocol with the actual intraoperative issues would be of great importance.

## 5. Conclusions

1. The surgical corridor of the transcanal suprageniculate approach is defined by its dimensions in two crucial checkpoints—the external and internal windows. The external window is set on a tilted horizontal plane and it is limited by the middle cranial fossa anterolaterally and a set of structures such as the proximal tympanic facial nerve, the ampulla of the anterior semicircular canal, the vestibule and the geniculate ganglion. The internal window is set on a parasagittal plane passing through the tympanic facial nerve and it is limited by the vestibule and the ampulla of the anterior semicircular canal posteriorly and the middle cranial fossa anteriorly.
2. The dimensions of the external and internal windows decrease in a posterior to anterior direction. It corresponds to the slope of the middle cranial fossa that is found anteriorly to the anterior semicircular canal. The width of the external window and the height of the internal window positively correlate with each other. These measurements do not seem to be correlated to side and gender.
3. The suprageniculate pneumatization degree may be classified into three types. Type A means no pneumatization in the area of the geniculate ganglion. Type B means pneumatization of the air cells above the geniculate ganglion. Type C means pneumatization above the geniculate ganglion that extends to the area of the petrous apex. This classification might help with the preoperative assessment of the area of the suprageniculate fossa as it correlates with the width and height of the surgical windows of the suprageniculate approach.

## Figures and Tables

**Table 1:** Basic descriptive statistics of the studied variables according to sides.

	M	SD	Sk	Min	Max	*p*
Left side						
Tilted Axial Plane Wp	7.52	1.91	0.60	4.02	13.00	0.066
Tilted Axial Plane Wm	5.63	2.30	0.41	0.00	11.30	0.206
Tilted Axial Plane Wa	1.76	1.56	0.68	0.00	6.74	<0.001
Parasagittal Plane Hp	7.67	1.34	−0.30	4.28	11.20	0.146
Parasagittal Plane Hm	4.56	1.60	0.25	0.00	9.69	0.399
Parasagittal Plane Ha	1.83	1.79	1.74	0.00	9.65	<0.001
Tilted Axial Plane D1	7.17	1.79	0.47	3.29	12.70	0.179
Parasagittal Plane D2	7.17	1.80	0.51	3.61	12.80	0.124
Right side						
Tilted Axial Plane Wp	7.40	1.97	0.55	3.06	14.00	0.190
Tilted Axial Plane Wm	5.60	2.43	0.18	0.00	11.40	0.816
Tilted Axial Plane Wa	1.78	1.73	1.61	0.00	8.48	<0.001
Parasagittal Plane Hp	7.56	1.17	0.10	5.28	10.00	0.302
Parasagittal Plane Hm	4.45	1.37	−0.07	1.51	7.42	0.517
Parasagittal Plane Ha	1.56	1.28	0.52	0.00	5.18	<0.001
Tilted Axial Plane D1	7.43	1.80	0.24	3.68	12.50	0.413
Parasagittal Plane D2	7.47	1.83	0.23	3.59	12.60	0.566

M—mean value; SD—standard deviation; Sk—skewness; Min and Max—the lowest and highest value; *p*—*p*-value.

**Table 2:** Height and width of the corridor and patients’ gender.

	Female (n = 40)		Male (n = 40)		*p*	Cohen’s d
	M	SD	M	SD		
Left side						
Tilted Axial Plane Wp	7.49	1.97	7.55	1.87	0.890	0.03
Tilted Axial Plane Wm	5.43	2.20	5.83	2.41	0.439	0.17
Tilted Axial Plane Wa	1.64	1.57	1.88	1.55	0.505	0.15
Parasagittal Plane Hp	7.47	1.26	7.87	1.39	0.176	0.31
Parasagittal Plane Hm	4.21	1.18	4.91	1.88	0.051	0.44
Parasagittal Plane Ha	1.47	1.21	2.18	2.19	0.078	0.40
Tilted Axial Plane D1	6.86	1.45	7.48	2.05	0.124	0.35
Parasagittal Plane D2	6.87	1.49	7.47	2.04	0.132	0.34
Right side						
Tilted Axial Plane Wp	7.12	2.01	7.69	1.92	0.199	0.29
Tilted Axial Plane Wm	5.28	2.16	5.91	2.66	0.248	0.26
Tilted Axial Plane Wa	1.52	1.62	2.04	1.81	0.175	0.31
Parasagittal Plane Hp	7.38	1.21	7.75	1.10	0.156	0.32
Parasagittal Plane Hm	4.32	1.33	4.58	1.42	0.388	0.19
Parasagittal Plane Ha	1.42	1.23	1.71	1.33	0.308	0.23
Tilted Axial Plane D1	6.90	1.60	7.97	1.85	**0.007**	0.62
Parasagittal Plane D2	6.95	1.61	7.98	1.90	**0.010**	0.59

M—mean value; SD—standard deviation; *p*—*p*-value; d—Cohen’s d. Bold numbers are the statistically significant ones.

**Table 3:** Corridor’s height and width and measurement side.

	Left Side		Right Side		*p*	Cohen’s d
	M	SD	M	SD		
Tilted Axial Plane Wp	7.52	1.91	7.40	1.97	0.628	0.05
Tilted Axial Plane Wm	5.63	2.30	5.60	2.43	0.918	0.01
Tilted Axial Plane Wa	1.76	1.56	1.78	1.73	0.913	0.01
Parasagittal Plane Hp	7.67	1.34	7.56	1.17	0.373	0.10
Parasagittal Plane Hm	4.56	1.60	4.45	1.37	0.517	0.07
Parasagittal Plane Ha	1.83	1.79	1.56	1.28	0.179	0.15
Tilted Axial Plane D1	7.17	1.79	7.43	1.80	0.240	0.13
Parasagittal Plane D2	7.17	1.80	7.47	1.83	0.191	0.15

M—mean value; SD—standard deviation; *p*—*p*-value; d—Cohen’s d.

**Table 4:** Correlation between the different height and width measurements of the surgical corridor.

**Left Side**								
		**Tilted Axial Plane Wp**	**Tilted Axial Plane Wm**	**Tilted Axial Plane Wa**	**Parasagittal Plane Hp**	**Parasagittal Plane Hm**	**Parasagittal Plane Ha**	**Tilted Axial Plane D1**
Tilted Axial Plane Wm	r Pearson	**0.60**						
	significance	**<0.001**						
Tilted Axial Plane Wa	r Pearson	**0.32**	**0.42**					
	significance	**0.004**	**<0.001**					
Parasagittal Plane Hp	r Pearson	0.09	0.21	**0.35**				
	significance	0.421	0.060	**0.002**				
Parasagittal Plane Hm	r Pearson	0.12	**0.38**	**0.50**	**0.62**			
	significance	0.284	**<0.001**	**<0.001**	**<0.001**			
Parasagittal Plane Ha	r Pearson	**0.22**	**0.33**	**0.75**	**0.38**	**0.64**		
	significance	**0.050**	**0.003**	**<0.001**	**<0.001**	**<0.001**		
Tilted Axial Plane D1	r Pearson	**0.27**	**0.38**	**0.67**	**0.31**	**0.47**	**0.58**	
	significance	**0.016**	**<0.001**	**<0.001**	**0.005**	**<0.001**	**<0.001**	
Parasagittal Plane D2	r Pearson	**0.26**	**0.38**	**0.68**	**0.31**	**0.49**	**0.61**	**0.99**
	significance	**0.018**	**<0.001**	**<0.001**	**0.005**	**<0.001**	**<0.001**	**<0.001**
**Right side**								
		**Tilted Axial Plane Wp**	**Tilted Axial Plane Wm**	**Tilted Axial Plane Wa**	**Parasagittal Plane Hp**	**Parasagittal Plane Hm**	**Parasagittal Plane Ha**	**Tilted Axial Plane D1**
Tilted Axial Plane Wm	r Pearson	**0.72**						
	significance	**<0.001**						
Tilted Axial Plane Wa	r Pearson	**0.48**	**0.61**					
	significance	**<0.001**	**<0.001**					
Parasagittal Plane Hp	r Pearson	0.21	0.21	**0.32**				
	significance	0.064	0.063	**0.004**				
Parasagittal Plane Hm	r Pearson	0.23	0.27	**0.46**	0.77			
	significance	0.037	0.016	**<0.001**	<0.001			
Parasagittal Plane Ha	r Pearson	**0.33**	**0.42**	**0.72**	**0.37**	**0.57**		
	significance	**0.003**	**<0.001**	**<0.001**	**<0.001**	**<0.001**		
Tilted Axial Plane D1	r Pearson	**0.35**	**0.32**	**0.57**	**0.34**	**0.45**	**0.55**	
	significance	**0.001**	**0.003**	**<0.001**	**0.002**	**<0.001**	**<0.001**	
Parasagittal Plane D2	r Pearson	**0.35**	**0.33**	**0.57**	**0.34**	**0.45**	**0.56**	**1.00**
	significance	**0.002**	**0.003**	**<0.001**	**0.002**	**<0.001**	**<0.001**	**<0.001**

Different correlation intensities are depicted in different colors: yellow, weak strength (r < 0.3); orange, moderate strength (0.3 < r < 0.5); pink, high strength (0.5 < r < 0.7); red, very high strength (0.7< r < 0.9); burgundy, almost total or total correlation (r > 0.9). Bold numbers are the statistically significant ones.

**Table 5:** Correlation between the different access height and width measurements and the degree of pneumatization.

Pneumatization			
		Left Side	Right Side
Tilted Axial Plane Wp	Spearman’s rho	0.19	**0.39**
	significance	0.098	**<0.001**
Tilted Axial Plane Wm	Spearman’s rho	0.21	0.20
	significance	0.064	0.074
Tilted Axial Plane Wa	Spearman’s rho	**0.22**	**0.42**
	significance	**0.046**	**<0.001**
Parasagittal Plane Hp	Spearman’s rho	0.17	**0.26**
	significance	0.143	**0.022**
Parasagittal Plane Hm	Spearman’s rho	**0.51**	**0.50**
	significance	**<0.001**	**<0.001**
Parasagittal Plane Ha	Spearman’s rho	**0.24**	**0.39**
	significance	**0.030**	**<0.001**
Tilted Axial Plane D1	Spearman’s rho	**0.39**	**0.54**
	significance	**<0.001**	**<0.001**
Parasagittal Plane D2	Spearman’s rho	**0.39**	**0.55**
	significance	**<0.001**	**<0.001**

Different correlation intensities are depicted in different colors: yellow, weak strength (r < 0.3); orange, moderate strength (0.3 < r < 0.5); pink, high strength (0.5 < r < 0.7). Bold numbers are the statistically significant ones.

## Data Availability

The data belong to the Department of Dental and Maxillofacial Radiology, Medical University of Warsaw and are not available to share unless in the form included in the manuscript. Please contact authors for any further questions: Tomasz Wojciechowski’s email: tomasz.wojciechowski@wum.edu.pl.
